# Supplementary material for: Clinical characteristics of the “Gap” between the prevalence and incidence of hearing loss using National Health Insurance Service data
Source: PLoS One. 2024 Mar 8;19(3):e0299478. doi: 10.1371/journal.pone.0299478 (PMC10923459; doi:10.1371/journal.pone.0299478)
Supplement: S1 File — (DOCX) [file pone.0299478.s001.docx]

**Appendix**

Supplement 1. Prevalence by type of hearing loss (Number of patients per 100,000)

| **Type of HL** | **Year** | **2010** | **2011** | **2012** | **2013** | **2014** | **2015** | **2016** | **2017** | **2018** | **2019** | **2020** |
| --- | --- | --- | --- | --- | --- | --- | --- | --- | --- | --- | --- | --- |
| **Conductive** | Number of patients | 38,790 | 43,855 | 42,784 | 41,565 | 38,402 | 42,322 | 46,665 | 46,724 | 45,196 | 51,486 | 47,518 |
|  | Per 100,000 | 77.8 | 87.5 | 85.0 | 82.2 | 75.6 | 83.1 | 91.3 | 91.2 | 88.1 | 100.3 | 92.5 |
|  | Age (%) |  |  |  |  |  |  |  |  |  |  |  |
|  | <10 | 85.0 | 100.2 | 88.1 | 82.1 | 68.8 | 69.2 | 76.0 | 67.0 | 60.0 | 93.2 | 60.9 |
|  | 10-19 | 72.6 | 87.6 | 80.1 | 75.8 | 67.8 | 71.1 | 79.9 | 79.2 | 70.1 | 77.0 | 66.9 |
|  | 20-29 | 58.6 | 72.3 | 73.7 | 74.1 | 68.2 | 77.2 | 86.7 | 87.7 | 79.2 | 82.7 | 80.7 |
|  | 30-39 | 60.6 | 70.2 | 71.6 | 68.2 | 63.9 | 69.9 | 78.0 | 79.1 | 77.0 | 80.1 | 77.2 |
|  | 40-49 | 68.2 | 74.1 | 73.2 | 70.7 | 66.2 | 71.6 | 77.3 | 75.9 | 73.7 | 78.1 | 75.4 |
|  | 50-59 | 90.3 | 97.4 | 97.2 | 92.6 | 85.8 | 94.1 | 98.9 | 100.2 | 98.0 | 111.1 | 101.5 |
|  | ≥60 | 114.2 | 117.2 | 111.0 | 108.9 | 100.9 | 113.7 | 124.6 | 124.7 | 124.9 | 144.7 | 134.7 |
|  | Gender (%) |  |  |  |  |  |  |  |  |  |  |  |
|  | Male | 70.2 | 79.3 | 76.3 | 73.1 | 67.4 | 74.3 | 81.0 | 81.3 | 78.6 | 89.1 | 82.3 |
|  | Female | 85.4 | 94.7 | 93.7 | 91.3 | 83.9 | 91.9 | 101.6 | 101.1 | 97.5 | 111.4 | 102.8 |
| **Sensorineural** | Number of patients | 338,880 | 353,628 | 355,702 | 365,885 | 372,208 | 400,991 | 449,907 | 470,856 | 499,503 | 548,667 | 527,781 |
|  | Per 100,000 | 679.4 | 705.7 | 706.5 | 723.7 | 733.2 | 787.0 | 880.2 | 919.1 | 973.7 | 1,068.7 | 1,027.8 |
|  | Age (%) |  |  |  |  |  |  |  |  |  |  |  |
|  | <10 | 226.3 | 235.0 | 227.3 | 226.3 | 224.6 | 197.8 | 213.2 | 212.7 | 204.7 | 217.7 | 174.3 |
|  | 10-19 | 266.4 | 299.3 | 297.1 | 301.3 | 308.0 | 301.5 | 325.4 | 337.6 | 318.8 | 335.3 | 332.8 |
|  | 20-29 | 308.6 | 350.5 | 352.6 | 369.4 | 382.5 | 390.2 | 414.9 | 448.0 | 445.4 | 469.7 | 500.9 |
|  | 30-39 | 351.9 | 366.8 | 379.4 | 388.9 | 399.6 | 406.0 | 443.4 | 466.8 | 479.2 | 535.8 | 551.9 |
|  | 40-49 | 480.7 | 492.7 | 481.3 | 484.3 | 488.9 | 503.2 | 534.0 | 543.0 | 543.7 | 596.4 | 604.5 |
|  | 50-59 | 894.2 | 892.2 | 855.2 | 840.2 | 828.0 | 871.4 | 905.5 | 903.8 | 926.8 | 973.1 | 933.3 |
|  | ≥60 | 2,084.2 | 2,074.1 | 2,036.4 | 2,046.6 | 2,009.7 | 2,179.7 | 2,457.6 | 2,501.3 | 2,644.3 | 2,826.8 | 2,536.4 |
|  | Gender (%) |  |  |  |  |  |  |  |  |  |  |  |
|  | Male | 623.6 | 635.5 | 641.8 | 655.7 | 660.7 | 710.9 | 804.5 | 838.4 | 887.7 | 978.1 | 947.9 |
|  | Female | 735.4 | 767.3 | 771.3 | 791.7 | 805.7 | 863.0 | 955.7 | 999.6 | 1,059.3 | 1,159.0 | 1,107.3 |
| **Mixed** | Number of patients | 48,619 | 51,536 | 55,464 | 58,117 | 62,525 | 69,068 | 72,597 | 71,568 | 71,410 | 77,656 | 76,889 |
|  | Per 100,000 | 97.5 | 102.8 | 110.2 | 114.9 | 123.2 | 135.6 | 142.0 | 139.7 | 139.2 | 151.3 | 149.7 |
|  | Age (%) |  |  |  |  |  |  |  |  |  |  |  |
|  | <10 | 36.6 | 40.4 | 38.2 | 43.9 | 51.3 | 47.8 | 37.2 | 30.4 | 27.3 | 34.1 | 23.9 |
|  | 10-19 | 46.3 | 59.1 | 60.4 | 61.9 | 64.6 | 68.8 | 67.8 | 69.6 | 61.9 | 66.0 | 61.7 |
|  | 20-29 | 53.9 | 62.1 | 68.3 | 71.4 | 78.8 | 86.1 | 91.0 | 88.9 | 87.5 | 91.3 | 94.2 |
|  | 30-39 | 61.8 | 66.9 | 76.4 | 78.5 | 87.2 | 92.9 | 96.1 | 96.2 | 95.1 | 102.7 | 107.4 |
|  | 40-49 | 78.0 | 81.4 | 85.1 | 87.5 | 93.0 | 100.1 | 103.6 | 99.6 | 97.5 | 105.2 | 108.0 |
|  | 50-59 | 131.1 | 131.6 | 138.7 | 138.5 | 145.5 | 154.2 | 153.6 | 144.7 | 139.5 | 151.1 | 147.2 |
|  | ≥60 | 254.8 | 249.2 | 260.6 | 268.9 | 276.3 | 309.1 | 328.9 | 319.2 | 317.0 | 333.5 | 315.2 |
|  | Gender (%) |  |  |  |  |  |  |  |  |  |  |  |
|  | Male | 85.9 | 88.4 | 94.8 | 99.4 | 106.0 | 118.4 | 125.8 | 122.5 | 122.1 | 131.5 | 130.3 |
|  | Female | 109.1 | 116.0 | 125.5 | 130.5 | 140.3 | 152.7 | 158.3 | 156.8 | 156.2 | 170.9 | 169.0 |
| **Ototoxicity** | Number of patients | 684 | 753 | 339 | 289 | 294 | 275 | 207 | 204 | 184 | 170 | 129 |
|  | Per 100,000 | 1.37 | 1.50 | 0.67 | 0.57 | 0.58 | 0.54 | 0.40 | 0.40 | 0.36 | 0.33 | 0.25 |
|  | Age (%) |  |  |  |  |  |  |  |  |  |  |  |
|  | <10 | 1.61 | 1.61 | 0.47 | 0.30 | 0.28 | 0.24 | 0.26 | 0.24 | 0.34 | 0.19 | 0.07 |
|  | 10-19 | 0.81 | 0.76 | 0.38 | 0.28 | 0.38 | 0.22 | 0.27 | 0.17 | 0.17 | 0.12 | 0.14 |
|  | 20-29 | 0.69 | 0.61 | 0.23 | 0.23 | 0.29 | 0.26 | 0.21 | 0.21 | 0.13 | 0.09 | 0.09 |
|  | 30-39 | 0.56 | 0.73 | 0.38 | 0.22 | 0.19 | 0.16 | 0.09 | 0.08 | 0.06 | 0.08 | 0.03 |
|  | 40-49 | 0.91 | 0.96 | 0.52 | 0.48 | 0.41 | 0.39 | 0.30 | 0.17 | 0.18 | 0.20 | 0.16 |
|  | 50-59 | 1.87 | 1.95 | 0.93 | 0.55 | 0.67 | 0.40 | 0.42 | 0.55 | 0.40 | 0.35 | 0.21 |
|  | ≥60 | 3.33 | 3.86 | 1.62 | 1.64 | 1.52 | 1.67 | 1.01 | 1.01 | 0.91 | 0.86 | 0.67 |
|  | Gender (%) |  |  |  |  |  |  |  |  |  |  |  |
|  | Male | 1.49 | 1.56 | 0.64 | 0.53 | 0.55 | 0.53 | 0.40 | 0.41 | 0.39 | 0.33 | 0.29 |
|  | Female | 1.25 | 1.43 | 0.71 | 0.61 | 0.61 | 0.55 | 0.41 | 0.39 | 0.33 | 0.33 | 0.21 |
| **Presbycusis** | Number of patients | 29,006 | 28,500 | 26,111 | 27,320 | 27,401 | 29,111 | 34,282 | 33,573 | 35,815 | 37,634 | 32,609 |
|  | Per 100,000 | 58.2 | 56.9 | 51.9 | 54.0 | 54.0 | 57.1 | 67.1 | 65.5 | 69.8 | 73.3 | 63.5 |
|  | Age (%) |  |  |  |  |  |  |  |  |  |  |  |
|  | <10 | 0.00 | 0.00 | 0.00 | 0.00 | 0.00 | 0.00 | 0.00 | 0.00 | 0.02 | 0.00 | 0.00 |
|  | 10-19 | 0.04 | 0.00 | 0.00 | 0.00 | 0.00 | 0.00 | 0.00 | 0.00 | 0.00 | 0.00 | 0.00 |
|  | 20-29 | 0.03 | 0.00 | 0.00 | 0.00 | 0.00 | 0.02 | 0.06 | 0.00 | 0.00 | 0.00 | 0.00 |
|  | 30-39 | 0.00 | 0.00 | 0.00 | 0.00 | 0.00 | 0.00 | 0.01 | 0.01 | 0.01 | 0.04 | 0.00 |
|  | 40-49 | 2.10 | 1.47 | 1.94 | 1.54 | 1.73 | 1.51 | 2.03 | 1.44 | 1.27 | 1.39 | 1.25 |
|  | 50-59 | 19.99 | 20.56 | 16.89 | 16.39 | 15.89 | 15.47 | 16.61 | 13.46 | 13.04 | 13.51 | 10.73 |
|  | ≥60 | 364.51 | 345.24 | 304.54 | 306.48 | 294.60 | 299.06 | 335.81 | 315.63 | 321.45 | 321.23 | 264.59 |
|  | Gender (%) |  |  |  |  |  |  |  |  |  |  |  |
|  | Male | 48.5 | 45.8 | 42.4 | 44.8 | 44.1 | 48.4 | 58.1 | 57.2 | 61.3 | 64.9 | 56.7 |
|  | Female | 67.9 | 67.3 | 61.3 | 63.3 | 63.8 | 65.8 | 76.1 | 73.8 | 78.3 | 81.7 | 70.3 |
| **Sudden** | Number of patients | 68,113 | 70,860 | 75,703 | 80,082 | 84,445 | 91,425 | 96,747 | 101,369 | 105,343 | 114,524 | 117,072 |
|  | Per 100,000 | 136.6 | 141.4 | 150.4 | 158.4 | 166.4 | 179.4 | 189.3 | 197.9 | 205.3 | 223.1 | 228.0 |
|  | Age (%) |  |  |  |  |  |  |  |  |  |  |  |
|  | <10 | 12.9 | 10.7 | 12.5 | 14.3 | 14.9 | 12.1 | 10.9 | 9.4 | 9.8 | 14.0 | 10.3 |
|  | 10-19 | 55.7 | 60.3 | 62.7 | 65.8 | 68.2 | 71.5 | 74.8 | 76.2 | 71.6 | 73.3 | 79.1 |
|  | 20-29 | 93.1 | 105.7 | 111.2 | 120.4 | 122.7 | 129.6 | 139.3 | 147.1 | 145.1 | 155.3 | 175.0 |
|  | 30-39 | 120.0 | 127.1 | 135.1 | 141.0 | 147.6 | 160.3 | 169.2 | 180.7 | 185.4 | 198.0 | 215.8 |
|  | 40-49 | 150.0 | 156.5 | 164.1 | 170.3 | 180.2 | 192.5 | 203.2 | 212.4 | 222.3 | 241.5 | 247.2 |
|  | 50-59 | 216.7 | 215.2 | 228.1 | 232.1 | 243.4 | 256.0 | 259.9 | 269.2 | 277.5 | 297.9 | 293.0 |
|  | ≥60 | 260.1 | 251.9 | 261.1 | 272.7 | 278.8 | 302.2 | 316.4 | 320.0 | 330.9 | 354.4 | 340.2 |
|  | Gender (%) |  |  |  |  |  |  |  |  |  |  |  |
|  | Male | 122.8 | 126.6 | 134.2 | 142.5 | 150.3 | 161.2 | 170.0 | 178.5 | 183.5 | 200.1 | 202.3 |
|  | Female | 150.4 | 154.5 | 166.6 | 174.3 | 182.4 | 197.7 | 208.5 | 217.2 | 227.1 | 246.0 | 253.5 |
| **Noise-induced** | Number of patients | 8,329 | 8,600 | 8,845 | 8,349 | 8,249 | 9,243 | 8,525 | 8,640 | 8,356 | 9,095 | 8,930 |
|  | Per 100,000 | 16.7 | 17.2 | 17.6 | 16.5 | 16.2 | 18.1 | 16.7 | 16.9 | 16.3 | 17.7 | 17.4 |
|  | Age (%) |  |  |  |  |  |  |  |  |  |  |  |
|  | <10 | 0.8 | 1.3 | 1.3 | 1.2 | 1.1 | 1.2 | 1.1 | 1.4 | 0.8 | 0.9 | 0.4 |
|  | 10-19 | 8.0 | 9.8 | 9.0 | 7.3 | 8.2 | 8.5 | 7.9 | 7.7 | 6.2 | 6.9 | 5.5 |
|  | 20-29 | 17.8 | 19.0 | 18.7 | 18.4 | 16.4 | 17.5 | 15.6 | 15.3 | 13.3 | 12.3 | 10.7 |
|  | 30-39 | 14.7 | 13.7 | 13.7 | 12.2 | 11.7 | 12.5 | 11.1 | 11.6 | 10.0 | 10.3 | 8.7 |
|  | 40-49 | 20.2 | 20.3 | 20.0 | 18.5 | 17.7 | 19.4 | 17.6 | 17.6 | 15.2 | 15.9 | 14.4 |
|  | 50-59 | 30.3 | 29.5 | 30.2 | 28.0 | 28.1 | 31.1 | 27.5 | 27.8 | 27.4 | 27.0 | 25.8 |
|  | ≥60 | 19.8 | 20.4 | 22.4 | 21.8 | 21.5 | 25.2 | 24.1 | 23.8 | 25.7 | 31.0 | 32.9 |
|  | Gender (%) |  |  |  |  |  |  |  |  |  |  |  |
|  | Male | 23.1 | 23.5 | 24.2 | 22.9 | 22.9 | 25.0 | 23.5 | 24.2 | 24.0 | 26.4 | 26.2 |
|  | Female | 10.3 | 10.6 | 10.9 | 10.1 | 9.6 | 11.3 | 9.9 | 9.5 | 8.6 | 9.1 | 8.6 |
| **Other** | Number of patients | 136,697 | 135,136 | 140,059 | 146,071 | 152,492 | 174,029 | 200,902 | 215,061 | 241,191 | 262,850 | 256,085 |
|  | Per 100,000 | 274.1 | 269.7 | 278.2 | 288.9 | 300.4 | 341.6 | 393.1 | 419.8 | 470.1 | 512.0 | 498.7 |
|  | Age (%) |  |  |  |  |  |  |  |  |  |  |  |
|  | <10 | 210.8 | 210.6 | 221.2 | 218.1 | 196.5 | 202.9 | 210.4 | 206.2 | 220.1 | 241.3 | 186.3 |
|  | 10-19 | 142.3 | 156.6 | 156.6 | 159.3 | 160.9 | 175.4 | 202.8 | 219.4 | 226.6 | 232.7 | 221.9 |
|  | 20-29 | 141.0 | 154.0 | 162.8 | 169.0 | 184.6 | 206.7 | 233.3 | 256.1 | 273.2 | 274.7 | 299.2 |
|  | 30-39 | 150.3 | 159.2 | 171.4 | 181.2 | 200.2 | 229.9 | 255.7 | 275.7 | 300.9 | 324.0 | 331.5 |
|  | 40-49 | 205.4 | 200.4 | 209.7 | 218.5 | 229.0 | 256.4 | 283.3 | 305.5 | 326.4 | 352.0 | 359.7 |
|  | 50-59 | 369.4 | 344.9 | 347.2 | 351.9 | 356.1 | 394.5 | 434.0 | 453.0 | 492.7 | 514.9 | 503.5 |
|  | ≥60 | 687.0 | 628.2 | 620.7 | 635.1 | 647.5 | 738.3 | 867.4 | 900.1 | 1,023.4 | 1,113.5 | 1,020.6 |
|  | Gender (%) |  |  |  |  |  |  |  |  |  |  |  |
|  | Male | 253.0 | 243.4 | 250.2 | 261.7 | 269.8 | 308.8 | 352.9 | 373.9 | 419.5 | 460.6 | 449.1 |
|  | Female | 295.1 | 292.6 | 306.3 | 316.1 | 331.0 | 374.2 | 433.1 | 465.6 | 520.6 | 563.2 | 548.1 |
