# Supplementary material for: Clinical characteristics of the “Gap” between the prevalence and incidence of hearing loss using National Health Insurance Service data
Source: PLoS One. 2024 Mar 8;19(3):e0299478. doi: 10.1371/journal.pone.0299478 (PMC10923459; doi:10.1371/journal.pone.0299478)
Supplement: S2 File — (DOCX) [file pone.0299478.s002.docx]

**Appendix**

Supplement 2. Incidence by type of hearing loss (Number of patients per 100,000)

| **Type of HL** | **Year** | **2011** | **2012** | **2013** | **2014** | **2015** | **2016** | **2017** | **2018** | **2019** | **2020** |
| --- | --- | --- | --- | --- | --- | --- | --- | --- | --- | --- | --- |
| **Conductive** | Number of patients | 40,884 | 39,662 | 38,593 | 35,616 | 39,739 | 43,902 | 43,707 | 42,230 | 47,851 | 42,641 |
|  | Per 100,000 | 81.6 | 78.8 | 76.3 | 70.2 | 78.0 | 85.9 | 85.3 | 82.3 | 93.2 | 83.0 |
|  | Age (%) |  |  |  |  |  |  |  |  |  |  |
|  | <10 | 90.0 | 77.8 | 72.7 | 59.2 | 61.6 | 68.1 | 59.1 | 54.0 | 84.4 | 47.7 |
|  | 10-19 | 80.5 | 73.0 | 69.1 | 62.3 | 65.5 | 74.5 | 72.5 | 63.7 | 69.9 | 58.4 |
|  | 20-29 | 69.5 | 70.1 | 70.5 | 64.8 | 74.0 | 83.2 | 83.9 | 75.0 | 78.4 | 75.6 |
|  | 30-39 | 66.8 | 68.4 | 65.2 | 61.3 | 67.2 | 75.4 | 76.2 | 73.7 | 76.1 | 72.7 |
|  | 40-49 | 69.5 | 68.8 | 66.5 | 62.1 | 67.9 | 73.3 | 72.1 | 70.0 | 73.3 | 69.6 |
|  | 50-59 | 91.6 | 90.1 | 86.1 | 79.6 | 88.5 | 92.5 | 93.6 | 91.8 | 103.2 | 90.4 |
|  | ≥60 | 107.8 | 101.9 | 100.0 | 92.7 | 106.1 | 116.4 | 115.5 | 115.6 | 133.6 | 119.3 |
|  | Gender (%) |  |  |  |  |  |  |  |  |  |  |
|  | Male | 73.7 | 70.4 | 67.7 | 62.1 | 69.6 | 76.0 | 75.8 | 73.4 | 82.8 | 73.8 |
|  | Female | 88.5 | 87.2 | 85.0 | 78.2 | 86.3 | 95.8 | 94.8 | 91.2 | 103.6 | 92.2 |
| **Sensorineural** | Number of patients | 318,445 | 319,096 | 327,742 | 332,984 | 362,708 | 404,092 | 420,423 | 445,094 | 486,096 | 459,706 |
|  | Per 100,000 | 635.5 | 633.8 | 648.2 | 656.0 | 711.9 | 790.6 | 820.6 | 867.6 | 946.9 | 895.3 |
|  | Age (%) |  |  |  |  |  |  |  |  |  |  |
|  | <10 | 195.7 | 188.5 | 185.3 | 182.6 | 160.7 | 175.6 | 172.1 | 163.2 | 173.2 | 134.8 |
|  | 10-19 | 269.3 | 265.8 | 265.0 | 272.9 | 273.0 | 294.7 | 305.6 | 287.2 | 302.8 | 301.8 |
|  | 20-29 | 330.3 | 331.7 | 345.6 | 359.1 | 368.9 | 391.9 | 422.8 | 419.4 | 442.8 | 472.8 |
|  | 30-39 | 344.0 | 356.3 | 363.3 | 373.4 | 384.9 | 420.2 | 442.8 | 453.9 | 506.1 | 519.6 |
|  | 40-49 | 456.9 | 444.7 | 446.2 | 452.3 | 469.5 | 498.6 | 505.7 | 506.2 | 554.3 | 558.3 |
|  | 50-59 | 811.9 | 775.6 | 761.8 | 751.8 | 798.9 | 826.4 | 820.6 | 843.8 | 882.7 | 838.9 |
|  | ≥60 | 1,827.8 | 1,786.0 | 1,799.5 | 1,757.6 | 1,931.5 | 2,155.1 | 2,176.6 | 2,299.1 | 2,438.9 | 2,126.5 |
|  | Gender (%) |  |  |  |  |  |  |  |  |  |  |
|  | Male | 571.7 | 576.2 | 587.6 | 590.8 | 643.7 | 722.3 | 747.7 | 789.7 | 865.4 | 823.1 |
|  | Female | 691.6 | 691.5 | 708.8 | 721.1 | 779.9 | 858.8 | 893.4 | 945.2 | 1,027.9 | 967.0 |
| **Mixed** | Number of patients | 48,663 | 52,188 | 54,370 | 58,372 | 63,850 | 66,785 | 65,928 | 65,967 | 72,031 | 70,715 |
|  | Per 100,000 | 97.1 | 103.7 | 107.5 | 115.0 | 125.3 | 130.7 | 128.7 | 128.6 | 140.3 | 137.7 |
|  | Age (%) |  |  |  |  |  |  |  |  |  |  |
|  | <10 | 37.4 | 34.7 | 39.6 | 47.5 | 40.3 | 32.6 | 26.2 | 24.2 | 30.7 | 19.1 |
|  | 10-19 | 56.5 | 56.7 | 57.7 | 60.0 | 63.6 | 62.6 | 65.2 | 56.9 | 62.0 | 57.7 |
|  | 20-29 | 60.2 | 66.1 | 68.4 | 75.8 | 82.2 | 86.8 | 84.8 | 83.9 | 87.7 | 90.6 |
|  | 30-39 | 64.3 | 73.2 | 74.5 | 83.2 | 88.0 | 91.2 | 91.5 | 90.9 | 98.5 | 102.8 |
|  | 40-49 | 77.3 | 80.8 | 83.0 | 88.0 | 93.4 | 97.0 | 93.7 | 92.1 | 99.8 | 102.0 |
|  | 50-59 | 123.7 | 130.6 | 129.2 | 135.7 | 142.5 | 141.4 | 133.2 | 129.8 | 141.5 | 136.7 |
|  | ≥60 | 232.8 | 242.1 | 249.3 | 254.0 | 283.3 | 297.4 | 288.6 | 286.9 | 302.8 | 283.1 |
|  | Gender (%) |  |  |  |  |  |  |  |  |  |  |
|  | Male | 83.4 | 89.1 | 93.0 | 98.8 | 109.4 | 115.9 | 113.0 | 112.7 | 122.2 | 119.7 |
|  | Female | 109.7 | 118.2 | 122.1 | 131.1 | 141.2 | 145.4 | 144.4 | 144.4 | 158.3 | 155.6 |
| **Ototoxicity** | Number of patients | 695 | 294 | 269 | 269 | 242 | 182 | 189 | 169 | 142 | 117 |
|  | Per 100,000 | 1.39 | 0.58 | 0.53 | 0.53 | 0.47 | 0.36 | 0.37 | 0.33 | 0.28 | 0.23 |
|  | Age (%) |  |  |  |  |  |  |  |  |  |  |
|  | <10 | 1.42 | 0.39 | 0.28 | 0.22 | 0.17 | 0.22 | 0.24 | 0.30 | 0.17 | 0.07 |
|  | 10-19 | 0.67 | 0.27 | 0.25 | 0.36 | 0.21 | 0.23 | 0.11 | 0.17 | 0.06 | 0.10 |
|  | 20-29 | 0.56 | 0.15 | 0.21 | 0.29 | 0.24 | 0.21 | 0.18 | 0.12 | 0.07 | 0.06 |
|  | 30-39 | 0.68 | 0.34 | 0.21 | 0.19 | 0.14 | 0.08 | 0.07 | 0.06 | 0.07 | 0.03 |
|  | 40-49 | 0.90 | 0.46 | 0.43 | 0.32 | 0.34 | 0.24 | 0.16 | 0.15 | 0.19 | 0.12 |
|  | 50-59 | 1.78 | 0.84 | 0.51 | 0.66 | 0.37 | 0.40 | 0.54 | 0.38 | 0.29 | 0.21 |
|  | ≥60 | 3.63 | 1.44 | 1.55 | 1.38 | 1.46 | 0.87 | 0.94 | 0.84 | 0.72 | 0.63 |
|  | Gender (%) |  |  |  |  |  |  |  |  |  |  |
|  | Male | 1.41 | 0.55 | 0.49 | 0.49 | 0.46 | 0.36 | 0.38 | 0.36 | 0.29 | 0.25 |
|  | Female | 1.34 | 0.62 | 0.57 | 0.57 | 0.49 | 0.35 | 0.35 | 0.30 | 0.27 | 0.33 |
| **Presbycusis** | Number of patients | 26,791 | 24,363 | 25,625 | 25,482 | 27,055 | 32,040 | 30,939 | 32,935 | 34,466 | 29,193 |
|  | Per 100,000 | 53.5 | 48.4 | 50.7 | 50.2 | 53.1 | 62.7 | 60.4 | 64.2 | 67.1 | 56.9 |
|  | Age (%) |  |  |  |  |  |  |  |  |  |  |
|  | <10 | 0.00 | 0.00 | 0.00 | 0.00 | 0.00 | 0.00 | 0.00 | 0.02 | 0.00 | 0.00 |
|  | 10-19 | 0.00 | 0.00 | 0.00 | 0.00 | 0.00 | 0.00 | 0.00 | 0.00 | 0.00 | 0.00 |
|  | 20-29 | 0.00 | 0.00 | 0.00 | 0.00 | 0.02 | 0.06 | 0.00 | 0.00 | 0.00 | 0.00 |
|  | 30-39 | 0.00 | 0.00 | 0.00 | 0.00 | 0.00 | 0.01 | 0.01 | 0.01 | 0.03 | 0.00 |
|  | 40-49 | 1.42 | 1.91 | 1.51 | 1.70 | 1.50 | 2.02 | 1.42 | 1.24 | 1.38 | 1.20 |
|  | 50-59 | 20.22 | 16.46 | 15.94 | 15.46 | 14.97 | 16.05 | 12.95 | 12.56 | 13.07 | 10.22 |
|  | ≥60 | 323.67 | 283.39 | 286.88 | 273.24 | 277.33 | 313.29 | 290.33 | 295.10 | 293.60 | 236.37 |
|  | Gender (%) |  |  |  |  |  |  |  |  |  |  |
|  | Male | 43.2 | 39.7 | 42.0 | 41.2 | 45.1 | 54.4 | 53.0 | 56.6 | 59.7 | 50.8 |
|  | Female | 63.1 | 57.1 | 59.3 | 59.2 | 61.1 | 71.0 | 67.7 | 71.8 | 74.5 | 62.9 |
| **Sudden** | Number of patients | 65,699 | 69,421 | 72,979 | 76,588 | 82,691 | 87,153 | 91,100 | 94,273 | 102,257 | 104,203 |
|  | Per 100,000 | 131.1 | 137.9 | 144.3 | 150.9 | 162.3 | 170.5 | 177.8 | 183.8 | 199.2 | 202.9 |
|  | Age (%) |  |  |  |  |  |  |  |  |  |  |
|  | <10 | 10.0 | 11.4 | 12.8 | 13.5 | 10.5 | 9.8 | 8.7 | 9.2 | 13.0 | 9.6 |
|  | 10-19 | 56.4 | 58.2 | 60.5 | 62.4 | 65.4 | 68.9 | 69.8 | 65.9 | 67.3 | 73.4 |
|  | 20-29 | 100.6 | 104.9 | 113.3 | 115.1 | 121.2 | 130.4 | 137.1 | 134.8 | 144.3 | 163.3 |
|  | 30-39 | 120.1 | 126.1 | 131.8 | 137.3 | 149.1 | 156.3 | 167.0 | 170.3 | 182.7 | 199.1 |
|  | 40-49 | 145.7 | 150.5 | 155.7 | 164.5 | 174.7 | 185.0 | 193.2 | 200.6 | 217.3 | 222.9 |
|  | 50-59 | 197.2 | 208.0 | 209.7 | 218.1 | 229.5 | 231.6 | 239.5 | 246.4 | 262.7 | 258.2 |
|  | ≥60 | 229.8 | 235.4 | 243.5 | 248.0 | 268.1 | 278.2 | 280.3 | 289.2 | 309.5 | 293.0 |
|  | Gender (%) |  |  |  |  |  |  |  |  |  |  |
|  | Male | 117.4 | 122.9 | 130.0 | 136.5 | 145.8 | 153.3 | 160.4 | 163.7 | 178.4 | 179.9 |
|  | Female | 143.2 | 152.9 | 158.7 | 165.3 | 178.8 | 187.7 | 195.2 | 203.7 | 219.9 | 225.9 |
| **Noise-induced** | Number of patients | 8,260 | 8,357 | 7,890 | 7,792 | 8,786 | 7,992 | 8,094 | 7,826 | 8,569 | 8,356 |
|  | Per 100,000 | 16.5 | 16.6 | 15.6 | 15.3 | 17.2 | 15.6 | 15.8 | 15.3 | 16.7 | 16.3 |
|  | Age (%) |  |  |  |  |  |  |  |  |  |  |
|  | <10 | 1.2 | 1.3 | 1.2 | 1.0 | 1.1 | 1.1 | 1.4 | 0.8 | 0.9 | 0.4 |
|  | 10-19 | 9.6 | 8.8 | 7.1 | 8.0 | 8.3 | 7.6 | 7.5 | 6.0 | 6.7 | 5.3 |
|  | 20-29 | 18.4 | 17.8 | 17.6 | 15.8 | 16.9 | 15.0 | 14.7 | 12.8 | 11.8 | 10.2 |
|  | 30-39 | 13.3 | 13.1 | 11.7 | 11.3 | 12.1 | 10.7 | 10.9 | 9.5 | 9.8 | 8.4 |
|  | 40-49 | 19.5 | 19.0 | 17.6 | 16.9 | 18.4 | 16.5 | 16.4 | 14.2 | 15.1 | 13.5 |
|  | 50-59 | 28.2 | 28.4 | 26.3 | 26.4 | 29.4 | 25.6 | 25.9 | 25.5 | 25.1 | 23.8 |
|  | ≥60 | 19.2 | 20.7 | 20.1 | 19.5 | 23.6 | 22.1 | 22.1 | 23.8 | 29.2 | 30.8 |
|  | Gender (%) |  |  |  |  |  |  |  |  |  |  |
|  | Male | 22.5 | 22.9 | 21.6 | 21.6 | 23.7 | 22.0 | 22.6 | 22.4 | 24.9 | 24.5 |
|  | Female | 10.2 | 10.2 | 9.6 | 9.1 | 10.8 | 9.3 | 9.0 | 8.1 | 8.5 | 8.1 |
| **Other** | Number of patients | 123,794 | 128,331 | 133,424 | 139,990 | 160,548 | 184,283 | 196,130 | 220,249 | 238,279 | 229,717 |
|  | Per 100,000 | 247.0 | 254.9 | 263.9 | 275.8 | 315.1 | 360.5 | 382.8 | 429.3 | 464.1 | 447.4 |
|  | Age (%) |  |  |  |  |  |  |  |  |  |  |
|  | <10 | 181.0 | 188.2 | 182.9 | 162.4 | 170.7 | 175.5 | 172.0 | 184.1 | 201.8 | 145.9 |
|  | 10-19 | 140.3 | 139.6 | 142.3 | 145.0 | 158.6 | 185.0 | 198.4 | 204.7 | 206.6 | 197.2 |
|  | 20-29 | 145.5 | 153.5 | 159.0 | 175.4 | 196.1 | 220.4 | 242.3 | 257.8 | 256.7 | 280.6 |
|  | 30-39 | 151.0 | 162.3 | 171.5 | 191.0 | 218.9 | 243.0 | 261.3 | 284.7 | 304.9 | 312.2 |
|  | 40-49 | 187.2 | 196.1 | 204.5 | 215.2 | 240.6 | 265.1 | 284.9 | 303.6 | 327.6 | 333.3 |
|  | 50-59 | 315.7 | 319.7 | 323.1 | 328.9 | 366.5 | 399.1 | 414.8 | 453.9 | 469.7 | 458.2 |
|  | ≥60 | 572.4 | 565.6 | 575.7 | 588.2 | 675.6 | 787.9 | 809.2 | 922.9 | 997.5 | 896.0 |
|  | Gender (%) |  |  |  |  |  |  |  |  |  |  |
|  | Male | 222.6 | 228.6 | 238.2 | 247.3 | 284.6 | 323.3 | 341.0 | 382.7 | 417.1 | 401.9 |
|  | Female | 268.5 | 281.2 | 289.6 | 304.2 | 345.5 | 397.7 | 424.5 | 475.8 | 511.0 | 492.6 |
