# Supplementary material for: Clinical characteristics of the “Gap” between the prevalence and incidence of hearing loss using National Health Insurance Service data
Source: PLoS One. 2024 Mar 8;19(3):e0299478. doi: 10.1371/journal.pone.0299478 (PMC10923459; doi:10.1371/journal.pone.0299478)
Supplement: S3 File — (DOCX) [file pone.0299478.s003.docx]

**Appendix**

Supplement 3. Gap by type of hearing loss (Number of patients per 100,000)

| **Type of HL** | **Year** | **2011** | **2012** | **2013** | **2014** | **2015** | **2016** | **2017** | **2018** | **2019** | **2020** |
| --- | --- | --- | --- | --- | --- | --- | --- | --- | --- | --- | --- |
| **Conductive** | Number of patients | 2,971 | 3,122 | 2,972 | 2,786 | 2,583 | 2,763 | 3,017 | 2,966 | 3,635 | 4,877 |
|  | Per 100,000 | 5.9 | 6.2 | 5.9 | 5.5 | 5.1 | 5.4 | 5.9 | 5.8 | 7.1 | 9.5 |
|  | Age (%) |  |  |  |  |  |  |  |  |  |  |
|  | <10 | 10.2 | 10.2 | 9.4 | 9.6 | 7.6 | 7.8 | 7.9 | 6.1 | 8.9 | 13.2 |
|  | 10-19 | 7.1 | 7.2 | 6.7 | 5.5 | 5.6 | 5.4 | 6.7 | 6.4 | 7.1 | 8.5 |
|  | 20-29 | 2.8 | 3.6 | 3.6 | 3.4 | 3.3 | 3.5 | 3.8 | 4.1 | 4.3 | 5.1 |
|  | 30-39 | 3.5 | 3.3 | 3.1 | 2.6 | 2.7 | 2.6 | 2.9 | 3.3 | 4.0 | 4.5 |
|  | 40-49 | 4.5 | 4.4 | 4.2 | 4.1 | 3.7 | 4.0 | 3.8 | 3.8 | 4.7 | 5.8 |
|  | 50-59 | 5.8 | 7.2 | 6.5 | 6.2 | 5.6 | 6.4 | 6.6 | 6.2 | 7.9 | 11.2 |
|  | ≥60 | 9.3 | 9.1 | 8.9 | 8.2 | 7.6 | 8.2 | 9.3 | 9.3 | 11.1 | 15.4 |
|  | Gender (%) |  |  |  |  |  |  |  |  |  |  |
|  | Male | 5.6 | 5.9 | 5.4 | 5.3 | 4.6 | 5.0 | 5.5 | 5.3 | 6.3 | 8.5 |
|  | Female | 6.2 | 6.5 | 6.4 | 5.7 | 5.5 | 5.8 | 6.3 | 6.3 | 7.9 | 10.5 |
| **Sensorineural** | Number of patients | 35,183 | 36,606 | 38,143 | 39,224 | 38,283 | 45,815 | 50,433 | 54,409 | 62,571 | 68,075 |
|  | Per 100,000 | 70.2 | 72.7 | 75.4 | 77.3 | 75.1 | 89.6 | 98.4 | 106.1 | 121.9 | 132.6 |
|  | Age (%) |  |  |  |  |  |  |  |  |  |  |
|  | <10 | 39.2 | 38.8 | 41.1 | 42.0 | 37.2 | 37.6 | 40.6 | 41.5 | 44.5 | 39.5 |
|  | 10-19 | 30.0 | 31.3 | 36.3 | 35.1 | 28.5 | 30.7 | 32.0 | 31.6 | 32.6 | 31.0 |
|  | 20-29 | 20.1 | 20.8 | 23.8 | 23.4 | 21.3 | 23.0 | 25.2 | 26.0 | 26.9 | 28.1 |
|  | 30-39 | 22.9 | 23.1 | 25.6 | 26.2 | 21.2 | 23.2 | 24.0 | 25.3 | 29.7 | 32.3 |
|  | 40-49 | 35.8 | 36.6 | 38.0 | 36.6 | 33.7 | 35.4 | 37.2 | 37.5 | 42.0 | 46.2 |
|  | 50-59 | 80.3 | 79.6 | 78.4 | 76.2 | 72.5 | 79.0 | 83.2 | 83.0 | 90.4 | 94.4 |
|  | ≥60 | 246.3 | 250.5 | 247.1 | 252.1 | 248.2 | 302.5 | 324.7 | 345.2 | 387.9 | 409.9 |
|  | Gender (%) |  |  |  |  |  |  |  |  |  |  |
|  | Male | 63.8 | 65.6 | 68.0 | 69.9 | 67.1 | 82.3 | 90.7 | 98.0 | 112.6 | 124.8 |
|  | Female | 75.7 | 79.8 | 82.9 | 84.7 | 83.1 | 97.0 | 106.2 | 114.1 | 131.1 | 140.3 |
| **Mixed** | Number of patients | 2,873 | 3,276 | 3,747 | 4,153 | 5,218 | 5,812 | 5,640 | 5,443 | 5,625 | 6,174 |
|  | Per 100,000 | 5.7 | 6.5 | 7.4 | 8.2 | 10.2 | 11.4 | 11.0 | 10.6 | 11.0 | 12.0 |
|  | Age (%) |  |  |  |  |  |  |  |  |  |  |
|  | <10 | 3.0 | 3.4 | 4.3 | 3.7 | 7.5 | 4.6 | 4.2 | 3.1 | 3.5 | 4.7 |
|  | 10-19 | 2.6 | 3.7 | 4.2 | 4.6 | 5.2 | 5.2 | 4.5 | 4.9 | 4.0 | 4.0 |
|  | 20-29 | 1.9 | 2.2 | 3.0 | 3.0 | 3.9 | 4.2 | 4.1 | 3.6 | 3.5 | 3.7 |
|  | 30-39 | 2.6 | 3.1 | 3.9 | 4.0 | 5.0 | 4.9 | 4.6 | 4.2 | 4.1 | 4.6 |
|  | 40-49 | 4.2 | 4.2 | 4.5 | 5.0 | 6.7 | 6.6 | 5.8 | 5.4 | 5.4 | 6.0 |
|  | 50-59 | 7.9 | 8.1 | 9.2 | 9.8 | 11.7 | 12.3 | 11.5 | 9.6 | 9.6 | 10.5 |
|  | ≥60 | 16.5 | 18.4 | 19.6 | 22.3 | 25.8 | 31.5 | 30.6 | 30.0 | 30.7 | 32.1 |
|  | Gender (%) |  |  |  |  |  |  |  |  |  |  |
|  | Male | 5.0 | 5.7 | 6.4 | 7.2 | 9.0 | 9.9 | 9.5 | 9.4 | 9.3 | 10.6 |
|  | Female | 6.3 | 7.3 | 8.4 | 9.2 | 11.5 | 12.8 | 12.5 | 11.9 | 12.6 | 13.4 |
| **Ototoxicity** | Number of patients | 58 | 45 | 20 | 25 | 33 | 25 | 15 | 15 | 28 | 12 |
|  | Per 100,000 | 0.12 | 0.09 | 0.04 | 0.05 | 0.06 | 0.05 | 0.03 | 0.03 | 0.05 | 0.02 |
|  | Age (%) |  |  |  |  |  |  |  |  |  |  |
|  | <10 | 0.19 | 0.09 | 0.02 | 0.07 | 0.07 | 0.04 | 0.00 | 0.05 | 0.02 | 0.00 |
|  | 10-19 | 0.09 | 0.11 | 0.03 | 0.02 | 0.02 | 0.04 | 0.06 | 0.00 | 0.06 | 0.04 |
|  | 20-29 | 0.04 | 0.08 | 0.02 | 0.00 | 0.02 | 0.00 | 0.03 | 0.01 | 0.01 | 0.03 |
|  | 30-39 | 0.05 | 0.04 | 0.01 | 0.00 | 0.01 | 0.01 | 0.01 | 0.00 | 0.01 | 0.00 |
|  | 40-49 | 0.07 | 0.06 | 0.05 | 0.09 | 0.05 | 0.06 | 0.01 | 0.02 | 0.01 | 0.04 |
|  | 50-59 | 0.17 | 0.09 | 0.04 | 0.01 | 0.04 | 0.02 | 0.01 | 0.02 | 0.06 | 0.00 |
|  | ≥60 | 0.23 | 0.17 | 0.09 | 0.14 | 0.22 | 0.13 | 0.07 | 0.07 | 0.14 | 0.04 |
|  | Gender (%) |  |  |  |  |  |  |  |  |  |  |
|  | Male | 0.14 | 0.09 | 0.04 | 0.06 | 0.06 | 0.04 | 0.03 | 0.03 | 0.05 | 0.04 |
|  | Female | 0.09 | 0.09 | 0.04 | 0.04 | 0.07 | 0.06 | 0.03 | 0.03 | 0.06 | 0.01 |
| **Presbycusis** | Number of patients | 1,709 | 1,748 | 1,695 | 1,919 | 2,056 | 2,242 | 2,634 | 2,880 | 3,168 | 3,416 |
|  | Per 100,000 | 3.4 | 3.5 | 3.4 | 3.8 | 4.0 | 4.4 | 5.1 | 5.6 | 6.2 | 6.7 |
|  | Age (%) |  |  |  |  |  |  |  |  |  |  |
|  | <10 | 0.00 | 0.00 | 0.00 | 0.00 | 0.00 | 0.00 | 0.00 | 0.00 | 0.00 | 0.00 |
|  | 10-19 | 0.00 | 0.00 | 0.00 | 0.00 | 0.00 | 0.00 | 0.00 | 0.00 | 0.00 | 0.00 |
|  | 20-29 | 0.00 | 0.00 | 0.00 | 0.00 | 0.00 | 0.00 | 0.00 | 0.00 | 0.00 | 0.00 |
|  | 30-39 | 0.00 | 0.00 | 0.00 | 0.00 | 0.00 | 0.00 | 0.00 | 0.00 | 0.01 | 0.00 |
|  | 40-49 | 0.05 | 0.03 | 0.03 | 0.02 | 0.01 | 0.01 | 0.01 | 0.04 | 0.01 | 0.05 |
|  | 50-59 | 0.33 | 0.42 | 0.45 | 0.42 | 0.50 | 0.56 | 0.50 | 0.47 | 0.45 | 0.50 |
|  | ≥60 | 21.58 | 21.15 | 19.60 | 21.35 | 21.73 | 22.52 | 25.30 | 26.35 | 27.63 | 28.22 |
|  | Gender (%) |  |  |  |  |  |  |  |  |  |  |
|  | Male | 2.6 | 2.8 | 2.7 | 3.0 | 3.3 | 3.7 | 4.2 | 4.7 | 5.2 | 5.9 |
|  | Female | 4.2 | 4.2 | 4.0 | 4.6 | 4.8 | 5.1 | 6.1 | 6.5 | 7.2 | 7.4 |
| **Sudden** | Number of patients | 5,161 | 6,282 | 7,103 | 7,857 | 8,734 | 9,594 | 10,269 | 11,070 | 12,267 | 12,869 |
|  | Per 100,000 | 10.3 | 12.5 | 14.0 | 15.5 | 17.1 | 18.8 | 20.0 | 21.6 | 23.9 | 25.1 |
|  | Age (%) |  |  |  |  |  |  |  |  |  |  |
|  | <10 | 0.7 | 1.1 | 1.5 | 1.4 | 1.6 | 1.1 | 0.8 | 0.5 | 0.9 | 0.7 |
|  | 10-19 | 3.8 | 4.5 | 5.3 | 5.8 | 6.1 | 6.0 | 6.4 | 5.7 | 6.0 | 5.7 |
|  | 20-29 | 5.1 | 6.3 | 7.1 | 7.6 | 8.4 | 8.9 | 10.0 | 10.3 | 11.0 | 11.7 |
|  | 30-39 | 6.9 | 9.0 | 9.2 | 10.2 | 11.2 | 12.9 | 13.7 | 15.1 | 15.3 | 16.7 |
|  | 40-49 | 10.8 | 13.6 | 14.6 | 15.8 | 17.8 | 18.2 | 19.2 | 21.6 | 24.1 | 24.3 |
|  | 50-59 | 17.9 | 20.2 | 22.4 | 25.4 | 26.5 | 28.3 | 29.7 | 31.1 | 35.3 | 34.9 |
|  | ≥60 | 22.2 | 25.7 | 29.2 | 30.9 | 34.1 | 38.1 | 39.7 | 41.8 | 44.9 | 47.2 |
|  | Gender (%) |  |  |  |  |  |  |  |  |  |  |
|  | Male | 9.3 | 11.3 | 12.6 | 13.8 | 15.4 | 16.6 | 18.1 | 19.8 | 21.7 | 22.5 |
|  | Female | 11.2 | 13.6 | 15.5 | 17.1 | 18.9 | 20.9 | 22.0 | 23.4 | 26.1 | 27.6 |
| **Noise-induced** | Number of patients | 340 | 488 | 459 | 457 | 457 | 533 | 546 | 530 | 526 | 574 |
|  | Per 100,000 | 0.7 | 1.0 | 0.9 | 0.9 | 0.9 | 1.0 | 1.1 | 1.0 | 1.0 | 1.1 |
|  | Age (%) |  |  |  |  |  |  |  |  |  |  |
|  | <10 | 0.04 | 0.00 | 0.00 | 0.02 | 0.04 | 0.04 | 0.02 | 0.02 | 0.00 | 0.02 |
|  | 10-19 | 0.16 | 0.21 | 0.19 | 0.16 | 0.21 | 0.25 | 0.22 | 0.21 | 0.16 | 0.18 |
|  | 20-29 | 0.65 | 0.84 | 0.81 | 0.64 | 0.66 | 0.60 | 0.61 | 0.56 | 0.55 | 0.52 |
|  | 30-39 | 0.36 | 0.63 | 0.49 | 0.42 | 0.38 | 0.37 | 0.68 | 0.47 | 0.42 | 0.35 |
|  | 40-49 | 0.76 | 1.07 | 0.89 | 0.72 | 0.96 | 1.14 | 1.20 | 0.99 | 0.86 | 0.99 |
|  | 50-59 | 1.31 | 1.83 | 1.71 | 1.70 | 1.68 | 1.90 | 1.93 | 1.91 | 2.00 | 2.03 |
|  | ≥60 | 1.19 | 1.68 | 1.70 | 1.95 | 1.61 | 1.98 | 1.73 | 1.87 | 1.85 | 2.09 |
|  | Gender (%) |  |  |  |  |  |  |  |  |  |  |
|  | Male | 0.9 | 1.3 | 1.3 | 1.3 | 1.3 | 1.5 | 1.6 | 1.6 | 1.5 | 1.7 |
|  | Female | 0.4 | 0.6 | 0.5 | 0.5 | 0.5 | 0.6 | 0.5 | 0.5 | 0.5 | 0.5 |
| **Other** | Number of patients | 11,342 | 11,728 | 12,647 | 12,502 | 13,481 | 16,619 | 18,931 | 20,942 | 24,571 | 26,368 |
|  | Per 100,000 | 22.6 | 23.3 | 25.0 | 24.6 | 26.5 | 32.5 | 37.0 | 40.8 | 47.9 | 51.4 |
|  | Age (%) |  |  |  |  |  |  |  |  |  |  |
|  | <10 | 29.6 | 33.0 | 35.3 | 34.1 | 32.2 | 34.9 | 34.3 | 36.0 | 39.5 | 40.4 |
|  | 10-19 | 16.3 | 16.9 | 17.1 | 15.9 | 16.8 | 17.8 | 21.1 | 21.9 | 26.1 | 24.6 |
|  | 20-29 | 8.6 | 9.4 | 9.9 | 9.2 | 10.6 | 12.8 | 13.8 | 15.4 | 18.0 | 18.7 |
|  | 30-39 | 8.2 | 9.2 | 9.8 | 9.2 | 11.0 | 12.7 | 14.3 | 16.2 | 19.0 | 19.4 |
|  | 40-49 | 13.2 | 13.6 | 14.1 | 13.8 | 15.7 | 18.2 | 20.6 | 22.8 | 24.4 | 26.5 |
|  | 50-59 | 29.2 | 27.4 | 28.8 | 27.3 | 28.0 | 34.8 | 38.2 | 38.8 | 45.1 | 45.3 |
|  | ≥60 | 55.7 | 55.1 | 59.3 | 59.4 | 62.7 | 79.5 | 90.9 | 100.4 | 116.0 | 124.5 |
|  | Gender (%) |  |  |  |  |  |  |  |  |  |  |
|  | Male | 20.8 | 21.5 | 23.5 | 22.5 | 24.2 | 29.6 | 32.9 | 36.9 | 43.5 | 47.2 |
|  | Female | 24.1 | 25.1 | 26.5 | 26.7 | 28.7 | 35.4 | 41.0 | 44.8 | 52.2 | 55.5 |
